# Advances and Obstacles in Using CRISPR/Cas9 Technology for Non-Coding RNA Gene Knockout in Human Mesenchymal Stromal Cells

**DOI:** 10.3390/ncrna9050049

**Published:** 2023-08-24

**Authors:** Nataliya Basalova, Maria Illarionova, Mariya Skryabina, Maksim Vigovskiy, Anastasia Tolstoluzhinskaya, Alexandra Primak, Elizaveta Chechekhina, Vadim Chechekhin, Maxim Karagyaur, Anastasia Efimenko

**Affiliations:** 1 Institute for Regenerative Medicine, Medical Research and Education Center, Lomonosov Moscow State University, 27/10, Lomonosovsky Ave., 119192 Moscow, Russia; natalia_ba@mail.ru (N.B.); vigovskiy_m.a@mail.ru (M.V.); a.luzh@yandex.ru (A.T.); 2 Faculty of Medicine, Lomonosov Moscow State University, 27/1, Lomonosovsky Ave., 119192 Moscow, Russia; mar729i63illar90@yandex.ru (M.I.); skrebbka@gmail.com (M.S.); primak.msu@mail.ru (A.P.); voynovaes.pharm@gmail.com (E.C.); v-chech@mail.ru (V.C.)

**Keywords:** ncRNA, miRNA, hsa-miR-21, hsa-miR-29c, genome editing, CRISPR/Cas9, SpCas9D10A, mesenchymal stromal cells, secretome, fibrosis

## Abstract

Non-coding RNA (ncRNAs) genes have attracted increasing attention in recent years due to their widespread involvement in physiological and pathological processes and regulatory networks. The study of the function and molecular partners of ncRNAs opens up opportunities for the early diagnosis and treatment of previously incurable diseases. However, the classical “loss-of-function” approach in ncRNA function analysis is challenged due to some specific issues. Here, we have studied the potency of two CRISPR/Cas9 variants, wild-type (SpCas9wt) and nickase (SpCas9D10A) programmable nucleases, for the editing of extended DNA sequences in human mesenchymal stromal cells (MSCs). Editing the genes of fibrosis-related hsa-miR-21-5p and hsa-miR-29c-3p, we have shown that a pair of SpCas9D10A molecules can effectively disrupt miRNA genes within the genomes of MSCs. This leads not only to a decrease in the level of knockout miRNA in MSCs and MSC-produced extracellular vesicles, but also to a change in cell physiology and the antifibrotic properties of the cell secretome. These changes correlate well with previously published data for the knockdown of certain miRNAs. The proposed approach can be used to knock out ncRNA genes within the genomes of MSCs or similar cell types in order to study their function in biological processes.

## 1. Introduction

Genes of non-coding RNA (ncRNAs) have attracted increased attention in fundamental and applied research in recent years due to a wide range of already shown or so far only supposed mechanisms of their participation in the regulation of the genome [1,2]. Most intriguing is the fact that the functions of most ncRNAs are still unknown [3,4], although they may be involved in the pathogenesis of a disease or considered as a potential therapeutic target or drug. The classical approaches to establishing the functions of individual molecules in biological systems are the inhibition of the target molecule, its exogenous administration or the modification of its structure [5,6,7]. The same approaches (overexpression, repression or sequence change) can be applied to study the functions and molecular partners of individual ncRNAs.

One of the simplest approaches to suppressing ncRNA expression is to use antagomirs [8]; however, this approach is characterized by a number of disadvantages: among them is the difficulty of delivering antagomirs to the distinguished biological objects. Moreover, there is difficulty in differentiating specific and nonspecific effects when loading extracellular vesicles (EVs), the main mediators of intercellular communication involving ncRNAs [9], with antagomirs under experimental conditions.

An alternative experimental approach to suppressing the expression of a gene (including ncRNA genes) or changing its sequence is CRISPR/Cas9-mediated genome editing [10], which introduces a permanent genome modification and stably alters the expression level or sequence of a particular protein or ncRNA in the edited cell line [11,12]. In addition, to our knowledge, the CRISPR/Cas9 system does not directly affect the production and properties of EVs (as opposed to the EV transfection procedure), so, it can be applied to study the functions of EVs or the molecules (proteins and ncRNAs) transported by them.

Previously, we have shown that the EV fraction of the secretome of human multipotent mesenchymal stromal cells (MSCs) has antifibrotic activity, and this phenomenon, at least in part, is due to the action of ncRNA, namely hsa-miR-21 and hsa-miR-29c, which was confirmed by their inhibition using antagomirs [13]. However, the observed effect required confirmation using alternative experimental approaches and further study of its mechanism. To do this, we decided to use a more reproducible and less controversial (than the use of antagomirs) approach—CRISPR/Cas9-mediated genome editing. The results of hsa-mir-21 and hsa-mir-29c CRISPR/Cas9-mediated gene editing were consistent [14] with the data obtained using antagomirs [13]. However, while developing the CRISPR/Cas9-mediated ncRNA gene editing technology for hsa-mir-21 and hsa-mir-29c gene knockout in the difficult-to-transfect cell line ASC52telo (immortalized mesenchymal stromal cell line), we obtained interesting and even unexpected methodological observations, which we intend to share here.

To knock out the hsa-mir-21 and hsa-mir-29c genes, we tested two CRISPR/Cas9 variants, namely wild-type (WT) SpCas9 nuclease and SpCas9D10A nickase [15]. The usage of paired WT SpCas9 nucleases caused independent and rather efficient (up to 50%) editing at all target sites within the ASC52telo genome, but without any deletion of the intersite fragments encoding the target ncRNAs. At the same time, according to the literature, this CRISPR/Cas9 modification ensures the excision of target DNA fragments in a range of cell lines (e.g., HEK-293T, HeLa, U2OS, murine embryonic stem cells) with high efficiency [16,17,18], which was also confirmed by our own data in the HEK-293T cell line.

The treatment of target cells with the SpCas9D10 nickase system allows the effective editing of miRNA genes within the genome of the ASC52telo cell line; however, the predominant outcomes are microdeletions and fragment multiplications within the miRNA genes, rather than the excision of DNA fragments between nicks. The most important outcome is that these microdeletions and fragment multiplications in miRNA genes alter pri-miRNA duplex formation and avert miRNA maturation, which reduces expression of target miRNAs and changes the antifibrotic activity of the EVs obtained from edited cells. Here, we discuss the features of the nuclease activity of the WT SpCas9 and SpCas9D10 nickase genome editing systems and possible mechanisms that may underlie the repression of hsa-miR-21 and hsa-miR-29c activity after the SpCas9D10 nickase-mediated editing of their genes in a difficult-to-transfect human MSC cell line.

## 2. Results

### 2.1. Wild-Type Cas9 Nuclease Is Found to Be Inefficient for the Deletion of Extended DNA Fragments in ASC52telo Cell Line

**Figure 1:**
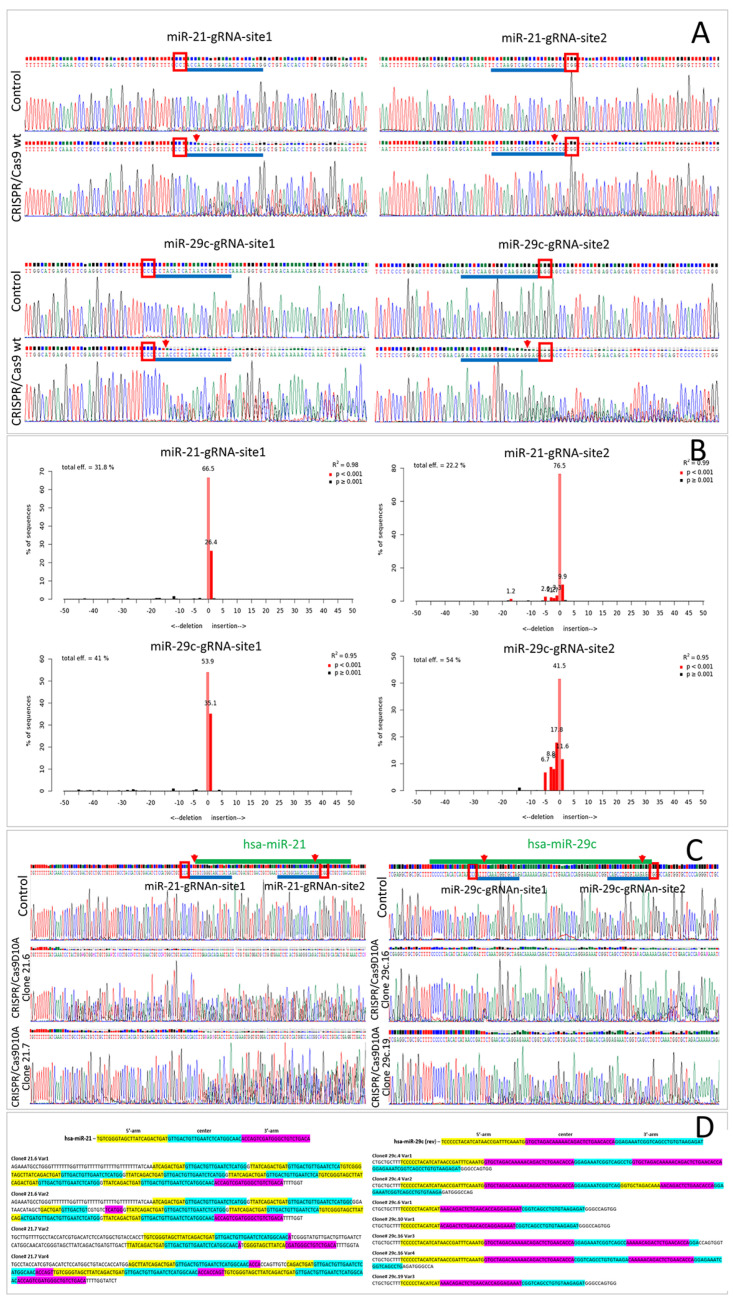
Results of genomic DNA sequencing within the edited regions of ASC52telo cells. (**A**)—genome editing using CRISPR/Cas9 wild-type system (indels are visible, but no deletion of the intersite DNA sequence occurs); (**B**)—the results of analysis of edited loci using TIDE (CRISPR/Cas9 wild type); (**C**)—genome editing using CRISPR/Cas9 nickase system: clones 21.6, 21.7, 29c.16 and 29c.19 (indels are visible; however, it is impossible to establish the exact sequences of individual DNA loci); (**D**)—sample sequences of individual DNA loci within the hsa-mir-21 and hsa-mir-29c genes of clones 21.6, 21.7, 29c.4, 29c.10, 29c.16 and 29c.19, edited with the CRISPR/Cas9D10A nickase system. Red boxes (**A**,**C**)—PAM-sequences; a blue line next to red box (**A**,**C**)—gRNA protospacer; red arrows (**A**,**C**)—intended cut sites; light red column with the number above it (**B**)—the percentage of intact alleles; dark red column with the number above it (**B**)—the percentage of modified alleles (insertions or deletions).

The goal of our study was to find a suitable CRISPR/Cas9 approach to knock out miRNA genes in our target cell line, ASC52telo. First, we tried to harness the paired WT SpCas9 (in LentiCRISPRv2GFP vector) accompanied by a pair of gRNAs to excise the DNA locus encoding the hsa-mir-21 or hsa-mir-29c gene. Sequencing of the edited loci and further analysis using Tracking of Indels by Decomposition (TIDE) revealed that the main outcome of genome editing in ASC52telo cells was short indels (≤5 nucleotides) at each of the double-strand break sites, with no intersite DNA fragment deletion (~500 bp) and with the intact allele variant predominating (Figure 1A,B). Despite moderate editing efficiency (up to 58%) at each of the sites, no excision of the target alleles was observed. We supposed that this may have been due to the independent and non-simultaneous editing at each of the sites.

At the same time, we found that the SpCas9 wild-type editing system, accompanied by a pair of gRNAs, exhibited high accuracy and efficiency while deleting a part of the pIX gene in HEK-293T cells—this means that WT SpCas9 was active and able to edit and excise DNA fragments (Figure S1). The results obtained in the ASC52telo and HEK293T cell lines were insufficient for a direct comparison of the efficiency of the WT SpCas9 and SpCas9D10A editing systems in these cell lines (different gRNA sequences, various distances between the cut sites)—therefore, any comparison was avoided.

As there was no excision in the WT SpCas9-treated ASC52telo population (Figure 1A), no individual clones of this population were obtained and studied. Since no signs of editing of the target miRNA genes using the CRISPR/Cas9 wild-type system in the ASC52telo cell line were observed, we studied the possibility of using the CRISPR/Cas9D10A nickase system for these tasks.

### 2.2. Cas9D10A Nickase Does Not Delete Extended DNA Fragments, but Disrupts miRNA Genes in ASC52telo Cell Line

Based on previously published data suggesting that two nicks in the opposed DNA strands separated by 40–70 bp can be converted into a double-strand break with further DNA fragment deletion [19], we decided to use the SpCas9D10A editing system for miRNA gene editing.

**Figure 2:**
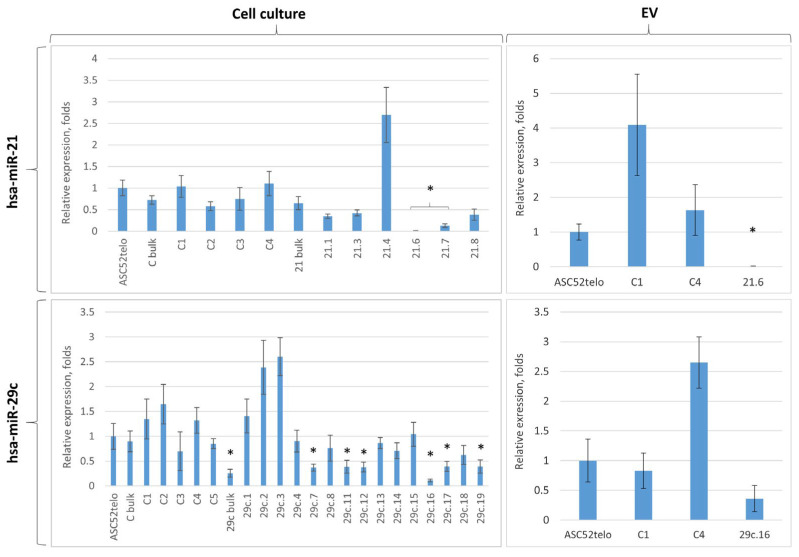
Relative expression levels (real-time PCR) of hsa-miR-21-5p and hsa-miR-29c-3p in native and CRISPR/Cas9-modified ASC52telo cells and their EV fractions; *n* ≥ 3. Groups: ASC52telo—native cell culture; C1, C2, etc.—clones treated with the CRISPR/Cas9 system in combination with scramble gRNAs; 21.x—clones treated with the CRISPR/Cas9 system in combination with guide RNAs to hsa-miR-29c; 29c.x—clones treated with the CRISPR/Cas9 system in combination with guide RNAs to hsa-miR-29c; bulk—cells with modification before cloning. * *p* < 0.05, compared to the appropriate control group (C bulk—for the **left panel**, ASC52 telo—for the **right panel**), *n* = 3, *t*-test.

Analyzing the sequencing results (by Sanger) of the SpCas9D10A-treated ASC52telo population, we found that the target miRNA genes (hsa-mir-21 and hsa-mir-29c) were severely altered; however, without cloning the obtained cell population, it was impossible to evaluate the editing outcome. Eight and nineteen ASC52telo cell clones were obtained for hsa-mir-21 and hsa-mir-29c, respectively. The level of hsa-miR-21-5p and hsa-miR-29c-3p was evaluated in some clones using qPCR, and, for the clones with a significant decrement in hsa-miR-21-5p and hsa-miR-29c-3p expression (Figure 2 and Table S1), the target region was sequenced (Figure 1C). Sanger sequencing of the edited region revealed that most of the target alleles were modified; however, the complexity of the obtained sequence did not allow us to establish the sequences of individual alleles (Figure 1C). To solve this problem, we obtained a library of plasmid vectors carrying individual variants of the edited alleles, ten for each ASC52telo cell clone obtained. Sequencing revealed 2–5 unique editing outcome variants (each of them represented 10% to 50% within the studied pool of sequences) for each ASC52telo clone (Table S1, Figures S2–S4). Figure 1D demonstrates examples of some of the sequences of the hsa-mir-21 and hsa-mir-29c gene alleles after genome editing, obtained from the sequencing results of the plasmid vector library.

In ASC52telo cell clones with the hsa-mir-29c gene edited, no intact alleles were detected (among 19 clones); however, some of the edited sequences (~5%) included an intact sequence of hsa-miR-29c (Table S1). In ASC52telo cell clones with the hsa-mir-21 gene edited, approximately 69% of the alleles remained intact (among eight clones studied, two had modifications in both alleles and one had a single allele modification) (Table S1).

Deletions of 9 to 27 nucleotides accompanied by microduplications within the edited region were the predominant outcomes (Figure 1C,D). Apparently, this occurred due to the presence of palindromic (homologic) sequences around the cut sites, which activated some microhomology-mediated DNA repair mechanisms (presumably microhomology-mediated repair (MHMR) or paired single-strand DNA repair (SSDR)).

Bioinformatics analysis revealed that the initial miRNA structures were disrupted and new duplex structures were formed. Most of them were processed by Drosha and Dicer, miRNA-processing enzymes, in an altered way, and some of them were no longer able to generate miRNAs (Table S1, Figures S2–S4).

### 2.3. Cas9D10A Nickase System Allows Us to Establish Biological Functions of Certain miRNAs in ASC52telo Cells

The analysis of target miRNA expression in the obtained ASC52telo cell lines revealed that the genome editing efficiency and outcomes varied from clone to clone. According to the real-time PCR results, no statistically significant difference in hsa-miR-21-5p expression was detected for clones 21.1, 21.3 and 21.8, compared to the control group (C bulk), but in clones 21.6 and 21.7, the expression of hsa-miR-21-5p decreased dramatically, 4000-fold and 7-fold, respectively, compared to the control group (C bulk): *p* < 0.05, *n* = 3, *t*-test (Figure 2, left panel).

For hsa-miR-29c-3p expression, no statistically significant difference was observed in clones 29c.4, 29c.8, 29c.13, 29c.14, 29c.15 and 29c.18, compared to the control group (C bulk). hsa-miR-29c-3p was reduced approximately three-fold in clones 29c.7, 29c.11, 29c.12, 29c.17 and 29c.19, compared to the control group (C bulk): *p* < 0.05, *n* = 3, *t*-test. The lowest expression of hsa-miR-29c-3p—seven-fold suppression, compared to the control ASC52telo group (C bulk)—was found in clone 29c.16: *p* < 0.05, *n* = 3, *t*-test (Figure 2, left panel).

According to the real-time PCR results, some of the clones (e.g., 21.4, 29c.2, 29c.3) had increased miRNA expression compared to the control ASC52telo cells (Figure 2, left panel). It is possible that this was due to a “duplication” of the pri-miRNA or a part of its sequence, recognized by the primers that we used for qPCR, after CRISPR/Cas9-mediated genome editing. Another possible explanation is that such mutated pri-miRNA can be processed to two or more miRNA, being inferior to an intact miRNA but able to bind the primers for qPCR.

Unfortunately, after genome editing, clones 29c.6 and 29c.10 stopped dividing and died; therefore, an insufficient cell mass for clones 29c.6 and 29c.10 was obtained for real-time PCR and functional analysis.

For the further analysis of the miRNA content in EVs, we chose one clone from each group with the most dramatic change in hsa-miR-21-5p or hsa-miR-29c-3p expression. We also selected two control clones treated with the CRISPR/Cas9 system in combination with the scramble gRNA (C1, C4), which had a hsa-miR-21-5p or hsa-miR-29c-3p expression level close to that in control ASC52telo cells. Real-time PCR of the EV content revealed similar changes in miRNA concentration in the fraction of EVs obtained from the conditioned medium of these clones, but a significant reduction in the miRNA content in ASC52telo-derived EVs was found only in 21.6 clones’ EVs (Figure 2, right panel).

**Figure 3:**
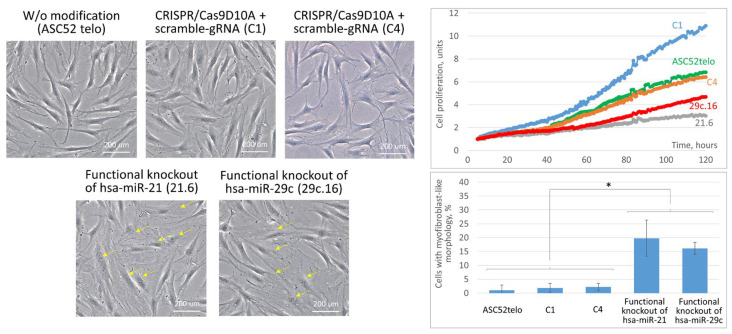
Characterization of the morphology and proliferation rate of ASC52telo cell cultures with the functional knockout of hsa-mir-21 and hsa-mir-29c genes. (**Left panel**)—Cell culture micrographs. Yellow arrows indicate cells with myofibroblast-like morphology. Phase contrast microscopy, 100×. (**Right panel top**)—Cell proliferation dynamics (IncuCyte Zoom). (**Right panel bottom**)—The percentage of cells with myofibroblast-like morphology. Clones C1 and C4 were treated with the CRISPR/Cas9D10A nickase system combined with the scramble gRNA. *—*p* < 0.05, *n* = 3.

Being edited, some ASC52telo clones showed significant changes in their morphology and proliferation rate. Thus, the progeny of clones 21.6 and 29c.16 contained an increased number of spread cells with a polygonal myofibroblast-like morphology and their proliferation was slowed (Figure 3). Disruption of the hsa-mir-29c gene reduced the cell proliferation rate by an average of two-fold and hsa-mir-21 by an average of three-fold. At the same time, clones treated with the CRISPR/Cas9 system in combination with scramble gRNA (C1, C4) revealed no changes in cell morphology (Figure 3). However, the C1 clone, unlike C4, had a high level of proliferation compared to the non-modified ASC52telo cell line (Figure 3). Because cell cultures with significantly different proliferation rates are difficult to synchronize to provide similar settings for conditioning and EV derivation, the C1 clone was formally excluded from further analysis. Taken together, the obtained data may indicate the important role of hsa-miR-21-5p and hsa-miR-29c-3p in maintaining the multipotent status of MSCs and counteracting spontaneous morphology changes in MSCs into myofibroblast-like cells, as well as in the processes of MSC proliferation, which, however, requires additional research.

EVs for the study of miRNA antifibrotic activity were collected from the edited clones C1, C4, 21.6 and 29c.19 and characterized to fit the properties of EVs (Figure S6).

We have previously shown that EVs from patient-derived MSCs and ASC52telo inhibit the TGF-beta-induced differentiation of fibroblasts into myofibroblasts, namely exerting antifibrotic activity [13,14]. Similarly, the antifibrotic effect of vesicular fractions obtained from ASC52telo cell cultures with the functional knockout of the hsa-mir-21 and hsa-mir-29c genes was studied in a model of TGFb-induced fibroblast differentiation in order to establish the functional effectiveness of the proposed approach in the genomic editing of miRNA genes.

**Figure 4:**
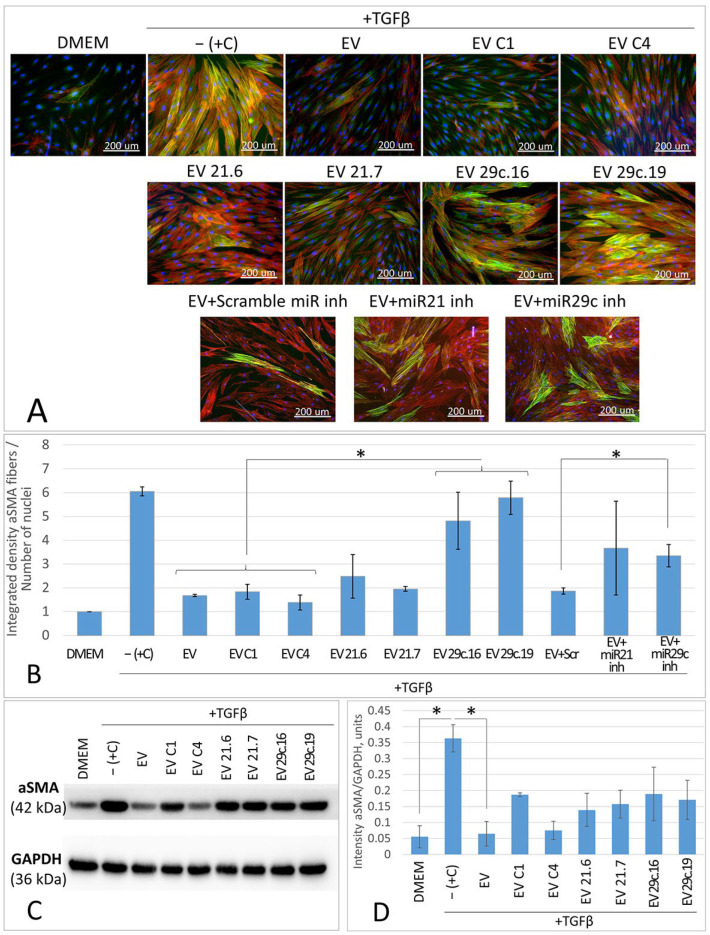
Content analysis of the myofibroblast marker alpha smooth muscle actin (aSMA) in the cultures of fibroblasts treated with TGFb combined with EV fraction obtained from native or CRISPR/Cas9-modified ASC52telo cells. (**A**)—Immunocytochemical staining: alpha smooth muscle actin—green, total actin (phalloidin)—red, DAPI—blue. (**B**)—Relative level of cell culture staining intensity (stain for the marker protein of myofibroblasts aSMA), *—*p* < 0.05, *n* ≥ 4. (**C**)—Western blot analysis. (**D**)—Quantitative analysis of Western blot results, normalized for GAPDH, *—*p* < 0.05, *n* ≥ 2.

We determined that EVs from clone 21.6 exerted reduced antifibrotic activity that led to the appearance of individual cells with a structured actin cytoskeleton, and the intensity of fiber staining for the myofibroblast marker alpha smooth muscle actin (aSMA) increased on average by 1.37-fold, although no statistically significant differences were observed (Figure 4A,B). Suppression of hsa-miR-29c-3p in EVs from clone 29c.16 led to an even greater increase in the number of cells with the structured actin cytoskeleton and to a 3.28-fold increase in the aSMA fiber staining intensity of the fibroblast cell culture (Figure 4A,B). These changes in fibroblast cells coincided with an increase in aSMA protein content (according to the results of Western blotting—Figure 4C,D) and with an increase in the average length of aSMA stress fibers (Figure S7).

Therefore, we have shown that the impairment of the sequence of the hsa-mir-21 and hsa-mir-29c genes in CRISPR/Cas9-modified ASC52telo cells decreases the antifibrotic activity of their EV fractions. The obtained results coincide with previously published data suggesting that the inhibitors (antagomirs) of hsa-miR-21-5p and hsa-miR-29c-3p decreased the antifibrotic activity of EVs and increased aSMA stress fiber formation in an in vitro model of fibrosis [13,14]. For a better comparison, these groups are shown in Figure 4A,B (samples “EV + miR21 inh” and “EV + miR29c inh”).

At the same time, EVs from ASC52telo clone C1 exposed to the CRISPR/Cas9 system in combination with the scramble gRNA inhibited fibroblast-to-myofibroblast differentiation in the same way as the EVs from native unmodified ASC52telo or MSCs.

## 3. Discussion

Recent studies have repeatedly demonstrated the important contribution of non-coding RNAs to the regulation of physiological and pathological processes in the cell and body [20,21]. Moreover, according to modern concepts, very few of the regulatory ncRNAs existing in the cell are known today, and, for known ncRNAs, their functions are often not established [22]. All this is complicated by the multifunctionality of ncRNAs, the degeneracy of ncRNA binding sites and the subtlety of genome regulation, which requires such studies to be carried out on primary cells or cell lines close to them. ncRNA overexpression, suppression or sequence changes are classical and convenient approaches to studying their molecular partners and biological functions within a cell. Here, as a proof-of-concept, we attempted to harness CRISPR/Cas9 modifications (SpCas9 wild type and SpCas9D10A nickase), able to excise continuous DNA fragments, for the knockout of non-coding RNA genes within the genomes of multipotent mesenchymal stromal cells (ASC52telo cell line). As a model for CRISPR/Cas9-mediated genome editing, we chose the hsa-mir-21 and hsa-mir-29c miRNA genes, with previously shown antifibrotic activity [13,14].

miRNAs are formed as a result of the processing of transcripts of their own genes (so called pri-miRNA) or pre-RNA introns of larger genes (so called mirtrons) [1,23]. miRNA maturation involves a number of RNA-processing enzymes (Drosha and Dicer, among them), which recognize the characteristic pri-mRNA hairpin structure and sequentially convert it into pre-miRNA and then into a miRNA/miRNA* duplex. The resulting miRNAs paired with proteins of the Argonaut family (Ago) are involved in a wide range of physiological and pathological processes through the regulation of the expression of individual genes. For more details on the mechanisms of miRNA biogenesis and maturation, see the review by O’Brien J. et al. [1].

Previously, it was shown that the WT CRISPR/Cas9 system generates one/several-nucleotide indels within the miRNA gene, disrupts maturation and changes the sequence of the final miRNA in HCT116 and HT-29 cells [24]. From our point of view, this approach is rather unpredictable and has restricted potency, because the frequency of indels, their lengths and the resulting sequence are largely determined by the nucleotide context and the type of edited cells [25].

The specificity of miRNA is determined by a short seed sequence of six to eight nucleotides [26]; however, it is a challenge to disrupt it, because a number of conditions must coincide [27,28,29]: the miRNA sequence must include PAM sites, the cut site must be within the seed sequence, the selected gRNAs must not be promiscuous and genomic alterations must be extended. The specificity and functions of other types of ncRNAs are also determined by extended sequences [30,31] and most single-nucleotide changes or frame shifts have little effect on their functions.

Indels are often small (1–3 nucleotides); they differ between the alleles and cannot drastically change the structure of the pri-miRNA, which retains its ability to mature and generate the modified miRNA [32]. Such modified miRNA may acquire new potential binding sites and new molecular partners, and this greatly complicates the study of its role in a particular biological process. This is why, in our opinion, WT SpCas9 combined with a single guide does not appear to be a reliable tool for the editing of extended sequences of ncRNAs.

From this point of view, approaches that allow us to cut out DNA fragments (WT CRISPR/Cas9 or CRISPR/Cas9D10A, both with a pair of gRNAs) [15,33] are more preferable for the removal or destruction of the miRNA gene or at least its seed sequence. Since the efficiency of various DNA repair mechanisms and, accordingly, the efficiency of genome editing may vary depending on the cell type, we considered both WT CRISPR/Cas9 (2 gRNA) and CRISPR/Cas9D10A in order to excise the hsa-mir-21 and hsa-mir-29c genes from the genomes of ASC52telo cells. Previously, we have shown the ability of these miRNAs to suppress fibrosis processes in in vitro and in vivo models, which was confirmed by blocking their activity using the corresponding inhibitors (antagomirs) [13]. The comparison of the previously obtained and present results could allow an evaluation of the effectiveness of the proposed approach for the editing of miRNA genes in order to establish their biological activity. We used a lentiviral delivery system because the difficult-to-transfect ASC52telo cell line was chosen as a model object.

As a result of editing the target genes, hsa-mir-21 and hsa-mir-29c, using the WT CRISPR/Cas9 system combined with a pair of gRNAs (the fragment to be deleted had approximately 500 bp), we observed independent editing at each of the four sites, while no excision of the target intersite DNA fragment occurred. Indels at each of the four edited sites did not allow us to confirm that the gRNAs used were inefficient and unable to provide genome editing—in some cases, the editing efficiency exceeded 50%. At the same time, it is known that several simultaneous DNA breaks with a high probability lead to the deletion/inversion of a DNA fragment, and the size of the deleted fragment can reach thousands and even millions of base pairs [16,17,18]. This was also confirmed by our own studies: the use of a similar editing system in HEK-293T cells revealed its high efficiency in the deletion of an extended DNA fragment (700 bp) with the “seamless” re-joining of the flanking DNA fragments (Figure S1). The peculiarity of all the studies mentioned above (those published and our own) is that they were carried out on aneuploid transformed cell lines, which, by their properties (e.g., transfection susceptibility, the level of expression of the genome editing system components, the level of DNA condensation and the activity of individual DNA repair systems), differ greatly from the studied ASC52telo cell line.

Direct comparisons in this case are inappropriate due to the differences in the cell cultures, gRNAs and the delivery systems used. However, we state that despite the efficient cleavage at all the target DNA sites, miRNA gene excision in wild-type CRISPR/Cas9-treated ASC52telo cells does not occur. It appears that each of these sites is edited independently and their editing is separated in time. A possible explanation for this may be the insufficient concentration of the Cas9 genome editor within the nucleus of an ASC52telo cell, which does not allow us to cut both target sites simultaneously (or with a difference of less than 1 h), but it ensures sequential cleavage with the following rapid NHEJ-mediated DNA repair at each of the sites.

Since, in this experiment, the CRISPR/Cas9 editing system was delivered as a genetic construct, the insufficient concentration of the editor may be explained either by the low activity of the expression cassette or by the relatively large volume of the mesenchymal stromal cells and the “dilution” of the synthesized Cas9 editor. This assumption is indirectly supported by the fact that the brightness of the LentiCRISPRv2GFP-transduced HEK293T and ASC52telo cells in the GFP channel varies dramatically (Cas9 and GFP are expressed from a single bicistronic mRNA) (our own data) and many published studies demonstrate that the promoter activity depends on the cell type [34,35].

Nevertheless, the effective excision of a DNA fragment in primary cells is possible, as confirmed by a number of studies in which genome editing was performed by introducing a one-time high peak concentration of RNP complexes guided by multiple gRNAs [36,37]. Apparently, the concentration of the double-strand breaking Cas9 genome editor and the method of its delivery play an important role in ensuring the efficient deletion of a DNA fragment. Other explanations are also possible, such as the difference in the activity of certain DNA repair systems in the HEK293T and ASC52telo cell lines [38,39], but all these assumptions need further confirmation.

The CRISPR/Cas9 system was delivered into ASC52 telo cells using lentiviral particles, which led to the integration of the cassette into the genomes of these cells. Therefore, the expression of the components of the editing system and genome editing lasted for a long time. This led to the repeated editing of the same site in dividing cells and to the emergence of more than two variants of alleles (the editing was continuous until the gRNA binding sites were destroyed). Studies of Cas9D10A nickase activity demonstrated that this CRISPR/Cas9 variant allowed the deletion of certain DNA fragments; however, contrary to our expectations, none of the clones studied had a complete deletion between the nicks (single-stranded DNA break), but only a partial deletion of one of the pri-miRNA arms, with the multiple self-reproduction of its fragment. We have not established the mechanism of this phenomenon; however, with a high degree of probability, it is due to the palindromic structure of the DNA sequence within the miRNA gene, which correlates with previously published data [40,41]. The reproducibility of the clone-to-clone editing outcome demonstrates that the same repair mechanism (presumably microhomology-mediated repair (MHMR) [42,43] is preferable when editing or damaging palindromic DNA sequences in ASC52 clones. We highlight the rather high efficiency of the nickase SpCas9D10A in impairing the target extended DNA sequence when using the same delivery and expression system (LentiCRISPRv2GFP). The insufficient Cas9 nickase concentration for simultaneous editing at both sites may be compensated for by the longer “lifetime” of a single-strand break compared to a double-strand DNA break, due to the insufficiency of base excision repair (BER) enzymes, which are the key players in nick repair [19,44].

We attempted to develop an approach for the deletion of ncRNA/miRNA genes, but, instead of deletion, we obtained the impaired miRNA gene sequence with a severe alteration to the 5′-/3′-arm or loop of the pri-miRNA. However, does this affect pri-miRNA duplex formation and miRNA maturation? According to the bioinformatics analysis, these alterations to the pri-miRNA sequence impair its duplex formation and subsequent miRNA maturation (Figures S2–S4); however, the obtained bioinformatics data need further experimental verification in a calibrated model system.

Sequence alterations and predicted impairments to pri-miRNA duplex formation were accompanied by a decrease in the concentrations of target miRNAs in the studied clones and a change in their functional activity. According to the real-time PCR, the concentrations of hsa-miR-21-5p and hsa-miR-29c-3p in the lysates of clones 21.6, 21.7, 29c.16 and 29c.19 and in EVs (clone 21.6) significantly decreased. The observed difference in the miRNA expression levels between certain clones was presumably due to clonal heterogeneity and different editing outcomes.

To study the functional changes of ASC52telo clones with the edited hsa-mir-21 and hsa-mir-29c genes, we performed a series of tests: we assessed the proliferation rate of CRISPR/Cas9-modified cells and the ability of their EVs to prevent the TGFb-induced fibroblast-to-myofibroblast differentiation.

The results of all these studies indicated that the content of functional hsa-miR-21-5p and hsa-miR-29c-3p in cells and EVs was reduced, which manifested itself in the 2–3-fold slowed proliferation of the CRISPR/Cas9-modified cells, as well as in a decrease in the antifibrotic activity of their EVs, more prominent for miR-29c-3p-edited cells. Several studies have demonstrated that miR-21 or miR-29c inhibition affects cell proliferation—for example, through the regulation of target proteins from the Ran GTPase family [13,45,46,47,48,49,50]. This coincides with our data describing the decline in the proliferation rate of MSCs with edited hsa-mir-21 and hsa-mir-29c genes. Moreover, two clones, with completely disrupted hsa-mir-29c genes, 29c.6 and 29c.10, even stopped dividing and died. We suppose that this may have been due to the deficiency of hsa-miR29c-3p.

All data obtained indicated that the concentration and function of the studied miRNAs in the edited clones were impaired, despite the fact that no complete deletion of the miRNA gene or its fragment occurred. We have not established a specific mechanism for this phenomenon, but we assume that a miRNA sequence defect impairs the formation of a proper hairpin-shaped structure, essential for Drosha/Dicer-mediated pri-miRNA processing, which prevents miRNA maturation (so called functional knockout).

This assumption is confirmed by the bioinformatics data obtained. The presence of a characteristic hairpin secondary structure and specific nucleotide sequences are the key conditions for the proper processing of pri-miRNA to pre-miRNA, pre-miRNA export to the cytoplasm and the subsequent processing of pre-miRNA to a miRNA/miRNA* duplex [51,52,53,54]. Severe alterations of the miRNA encoding DNA sequences disrupt the secondary structure and specific nucleotide sequences of pri-miRNA, which may lead to changes in the expression of downstream genes [55,56,57].

Thus, in this study, we aimed to harness CRISPR/Cas9 modifications (SpCas9 wild type and SpCas9D10A nickase), able to excise continuous DNA fragments, in order to remove or destroy an ncRNA gene in a primary cell line. As model objects, we used the hsa-mir-21 and hsa-mir29c genes within the genomes of ASC52telo cells. The use of Cas9D10A nickase made it possible to obtain cells with reduced expression of the target microRNAs, although, for the most part, not due to the deletion of its gene but due to the severe impairment of its sequence and further miRNA maturation. The data obtained allow us to state that this approach is suitable for the relatively simple and reproducible acquisition of cell lines based on human multipotent mesenchymal stromal cells with suppressed ncRNA/miRNA gene expression in order to study their functions.

## 4. Materials and Methods

### 4.1. Design and Assembly of Genetic Constructs

We chose the hsa-mir-21 (hsa-miR-21-5p) and hsa-mir-29c (hsa-miR-29c-3p) genes as model miRNA genes for disruption using the CRISPR/Cas9 system. According to our previously published data, these miRNAs play an important role in antifibrotic processes and mediate the antifibrotic effects of the secretomes of multipotent mesenchymal stromal cells (MSCs) and their immortalized cell line ASC52telo [13].

**Figure 5:**
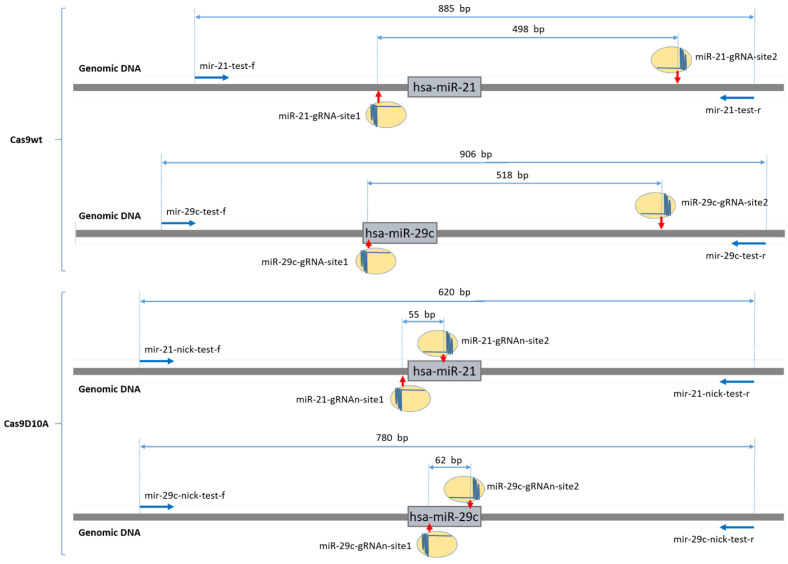
Layout of gRNA binding sites and primers relative to the hsa-mir-21 and hsa-mir-29c genes. Red arrows indicate sites of the expected DNA breaks. Blue bold arrows indicate the location and orientation of the primers used to amplify the edited DNA region within the miRNA gene.

The miRNA genes were modified using the CRISPR/Cas9 editing system: the LentiCRISPRv2GFP (Addgene, Watertown, MA, USA, #82416) vector for the WT SpCas9 and the LentiCRISPRD10Av2-GFP (obtained from LentiCRISPRv2GFP using site-directed mutagenesis) vector for the SpCas9D10A nickase variant. Pairs of guide RNAs (gRNA) were used to excise the hsa-mir-21 (https://mirbase.org, accessed on, 20 August 2023, #MI0000077) and hsa-mir-29c (https://mirbase.org, accessed on 20 August 2023, #MI0000735) genes. The gRNA sequences were selected as previously described [58]; gRNA specificity was assessed using the COSMID program [59]—gRNA protospacers without potential off-target sites within the exons of other genes were selected. The localization of gRNA binding sites relative to the hsa-mir-21 and hsa-mir-29c genes in the SpCas9 and SpCas9D10A experiments is demonstrated in Figure 5. The sequences of the oligonucleotides used are shown in Table 1 and Table 2 (double-strand break and nicking, respectively). Cloning of gRNA protospacers was performed according to the protocol of Feng Zhang et al. [60]. Scramble gRNA was encoded in control constructs. The sequences of the obtained genetic constructs were confirmed by Sanger sequencing. Obtained vectors were used for the assembly of lentiviral particles.

### 4.2. Cell Cultures

Cell lines used in the study: HEK-293T (ATCC, Manassas, VA, USA, #CRL-3-216) for lentiviral production, human MSCs (ASC52telo, ATCC, #/scrc-4000) for EV production, primary human dermal fibroblasts to assess the biological antifibrotic activity of MSC-produced EVs.

Human MSCs were cultured in AdvanceStem (HyClone, Logan, UT, USA) supplemented with 10% AdvanceStem supplement (HyClone) and 1% antibiotic–antimycotic (Gibco, Grand Island, NY, USA). HEK-293T cells were cultured in DMEM high-glucose (Gibco) supplemented with 10% fetal bovine serum (Gibco, Grand Island, NY, USA) and 1% antibiotic–antimycotic (Gibco). Dermal fibroblasts (DF) were obtained from the Biobank of the Institute for Regenerative Medicine, Medical Research and Educational Center, Lomonosov Moscow State University, collection ID: MSU_FB (https://human.depo.msu.ru, accessed on 20 August 2023). DF were cultured in DMEM low-glucose (Gibco) supplemented with 10% fetal bovine serum (Gibco) and 1% antibiotic–antimycotic (Gibco). All experiments were performed on fibroblasts no later than 12 passages. All cells were cultured at 5% CO_2_ and 37 °C.

### 4.3. Lentiviral Particle Assembly and Genetic Modification

The transfection of HEK-293T, assembly of lentiviral particles and transduction of ASC52telo were performed as previously described [61,62]. Here, 10% of the brightest GFP-positive cells were selected using the BD FACS Aria III cell sorter, and some of them were cloned in the wells of a 96-well plate. The cells were cultured at 37 °C in a CO_2_ incubator for approximately 4 weeks; medium drying was compensated for by adding fresh medium. Some of the obtained cell cultures, populations or clones were lysed for DNA sequencing, and some were used for secretome production and miRNA content analysis.

### 4.4. Analysis of the Genome Editing Efficiency

The editing efficiency of the hsa-mir-21 and hsa-mir-29c genes was assessed by the Sanger sequencing of PCR amplicons obtained from the edited region. Initially, the total cell population was analyzed, and PCR-mix-2-red (FBIS “Central Research Institute for Epidemiology”, #863) intended for complex template PCR amplification was used, according to the manufacturer’s instructions. Purified amplicons were sequenced by the Sanger method, and the results were analyzed using TIDE: Tracking of Indels by Decomposition [63].

For cell populations with effectively edited miRNA genes, the progeny of individual clones were analyzed. Phusion High-Fidelity PCR Master Mix with HF Buffer (ThermoFisher Scientific, Waltham, MA, USA, #F531L) was used to amplify the edited area within the genomes of individual clones, according to the manufacturer’s instructions. The obtained PCR amplicons were cloned into the pBluescript II SK (+) vector using the KpnI and EcoRI restriction sites, creating a library of sequences of the edited DNA locus for each of the clones. For each clone, 10 variants of the resulting genetic constructs were sequenced by the Sanger method (Figure 1). In order to evaluate the contribution of editing the hsa-mir21 and hsa-mir-29c loci to the formation of the corresponding pri-miRNA duplexes, we performed in silico folding using the “MFold” web server [64]. With the help of the deepMirCut software package [65], we identified the putative processing sites for Drosha- and Dicer-RNAses for the resulting duplexes, and a number of mature 5p- and 3p-miRNAs were predicted.

Clones with the most prominent destruction of the hsa-mir-21 and hsa-mir-29c genes were used for conditioned medium production.

### 4.5. Medium Conditioning

ASC52telo, native or CRISPR/Cas9-modified, were cultured under standard conditions until reaching 90% confluence. Afterwards, the cells were washed three times with Hank’s solution (PanEco, Moscow, Russia, #P020п) and incubated for 48 h in DMEM low-glucose medium (ThermoFischer Scientific, #11885084) supplemented with 1% antibiotic–antimycotic (Gibco). Then, the obtained medium was centrifuged for 10 min at 300 g to remove cell debris. The supernatant containing EVs was decanted and concentrated to 5-fold using Amicon centricons (300 kDa, Sartorius, Göttingen, Germany, #Vs3052). The concentrated conditioned medium enriched in EV fractions was used in the cellular model of fibrosis or stored at −80 °C.

### 4.6. PCR Analysis of miRNAs in Cell Culture and Extracellular Vesicles

Real-time PCR was used to evaluate the expression of the studied miRNAs in the ASC52telo cell culture and MSC-derived EVs (EV-MSCs). Cell cultures were washed with phosphate-buffered saline solution twice. Thereafter, cultures or concentrated EV-MSCs were lysed with commercial RLT+ buffer (Qiagen, Venlo, The Netherlands). Total RNA enriched with miRNA was isolated using the RNeasy Plus Mini Kit (Qiagen) according to the manufacturer’s protocol. For DNA removal, gDNA eliminator columns (Qiagen) were used. The concentration and purity of RNA in the obtained samples were determined by measuring A260/A230 and A260/280 using the Nanodrop-1000 (ThermoFisher Scientific). Samples with A260/280 from 1.9 to 2.1 were used for further analysis.

cDNA was synthesized using the miScript II RT Kit (Qiagen) according to the manufacturer’s protocol. Here, 10× miScript Nucleics Mix, 5× miScript HiFlex Buffer and miScript Reverse Transcriptase Mix were used to carry out the reaction. Reverse transcription was performed in a Mastercycler Nexus (Eppendorf, Hamburg, Germany) PCR amplifier with a hot lid for 1 h at 37 °C, followed by inactivation for 5 min at 95 °C.

The miRNA content was determined by real-time PCR using a commercial miScript SYBR Green PCR Kit (Qiagen) in a QuantStudio 5 Real-Time PCR System DNA Amplifier (ThermoFisher Scientific) using the following steps: initial denaturation for 15 min at 95 °C, followed by 50 cycles of amplification (denaturation at 94 °C for 15 s; annealing at 55 °C for 30 s; extension at 70 °C for 30 s). Oligodeoxynucleotides miR-21-5p and miR-29c-3p (Qiagen) and (RNU6, CGCAAGGATGACACGCAAAT, Evrogen, Moscow, Russia) were used as primers; miScript Universal Primer (Qiagen) was used as a reverse primer. The results obtained were normalized to the housekeeping gene RNU6.

### 4.7. Cellular Model of Fibrosis

To assess the effect of microRNAs within the EV-MSCs on fibroblast differentiation, an in vitro model of the TGFb-induced differentiation of fibroblasts into myofibroblasts was used. For this, primary human dermal fibroblasts (8–12 passages) were seeded in culture plates in complete growth medium at a rate of 15.000/cm^2^. After 24 h, the plates were washed with DMEM low-glucose (Gibco) and left in the second batch of DMEM low-glucose for overnight deprivation. After deprivation, appropriate solutions were added to the cells in each group to induce differentiation: negative control “DMEM” group—DMEM LG; positive control “+C” group—DMEM LG + 5 ng/mL TGFb; “EV” group—DMEM LG + 5 ng/mL TGFb + EV of native ASC52telo; “EV C1” and “EV C4” groups—DMEM LG + 5 ng/mL TGFb + ASC52telo EV treated with scramble gRNA (clones C1 and C4, respectively); “EV 21.6” and “EV 21.7” groups—DMEM LG + 5 ng/mL TGFb + EV ASC52telo with the impaired hsa-mir-21 gene (clones 21.6 and 21.7, respectively); “EV 29c.16” and “EV 29c.19” groups—DMEM LG + 5 ng/mL TGFb + EV ASC52telo with the impaired hsa-mir-29c gene (clones 29c.16 and 29c.19, respectively). After 4 days of incubation, the cells were used for further analysis.

### 4.8. Immunocytochemical Analysis

Cells were fixed with 4% paraformaldehyde in phosphate-buffered saline (PanEco, Moscow, Russia, #P060) for 10 min at room temperature and permeabilized with 0.1% Triton X-100 for 10 min. Non-specific binding was blocked with 10% normal goat serum (Abcam, Cambridge, UK) in 1% bovine serum albumin (PanEco) and cells were stained with the antibodies to alpha-actin (ab5694, Abcam), vinculin (v9264, Sigma, St. Louis, MO, USA) or non-specific rabbit IgG (NSC-2025; Santa Cruz Biotechnology, Dallas, TX, USA) overnight at 4 °C. Staining with secondary antibodies conjugated to Alexa 488 or 594 (#A11034, #A21203, Invitrogen, Waltham, MA, USA) was performed at room temperature in the dark for 1 h. The nuclei were stained with DAPI (D9542, Sigma). Images, at least 4 representative fields of view per well, were obtained using an inverted microscope with a fluorescent module and the Leica DMi8 and Leica DFC7000 T cameras (Leica Microsystems GmbH, Wetzlar, Germany), followed by processing with LasX (Leica Microsystems GmbH, Germany) and FIJI (GitHub Inc., San Francisco, CA, USA). To calculate the signal from aSMA stress fibers in the images, the background was subtracted using the Gaussian Blur and Subtract Background functions. To select the aSMA stress fiber zone, the obtained images were binarized using the Threshold function, after which the object’s area and integrated density were measured.

### 4.9. Western Blotting

ASC52telo cultures, native or CRISPR/Cas9-modified, were lysed in 2× Lamley’s buffer. The protein concentration in the lysate was determined using the microBCA protein assay method (Thermo Fisher Scientific, #23235). Electrophoresis in SDS-PAGE was carried out in a standard manner, and the samples were transferred to a PVDF membrane (Bio-Rad, Hercules, CA, USA, #1620177). Non-specific binding was blocked with 5% non-fat dry milk in 0.1% Tween 20 (Sigma, #P1379-250ML) on TBST for 1 h at room temperature and then blots were stained with the antibodies to alpha-actin (aSMA) (#ab32575, Abcam, Cambridge, UK) or GAPDH (Cell Signaling, Danvers, MA, USA, #2118) overnight at 4 °C. After washing in TBST, blots were incubated with secondary antibodies labeled with horseradish peroxidase (#P-RAM Iss, #P-RAQ Iss, Imtek, Moscow, Russia) for 1 h. Labeled proteins were visualized using the ECL kit (Pierce, Waltham, MA, USA) and the ChemiDocTM Touch imaging system (Bio-Rad Laboratories, Hercules, CA, USA).

### 4.10. Evaluation of the Proliferative Activity of ASC52telo Clones

The proliferative activity of ASC52telo clones, native or CRISPR/Cas9-modified, was assessed using the in vivo microscopy IncuCyte Zoom (Sartorius, Göttingen, Germany) system. For this, cell cultures were planted in culture plates in standard growth medium with confluence of 30%. The plate was set into the IncuCyte Zoom system: magnification 10×, shooting interval every 30 min. Each of the samples was analyzed in duplicate. After 7 days, when the cultures reached the confluent state, the acquisition was stopped and the automatic analysis of cell proliferation parameters was performed using the IncuCyte software (6–9 fields of view for each well).

### 4.11. Statistical Analysis

Statistical analysis was performed using the SigmaPlot11.0 software. Data were assessed for normality of distribution using the Kolmogorov–Smirnov test. Differences between groups were analyzed using ANOVA (Kruskal–Wallis test) or ANOVA on ranks (Dunn’s test), depending on whether data were normally distributed or not. For pairwise comparisons, a *t*-test was used. Data were expressed as the mean ± standard deviation or median (25%; 75%) depending on the test used. We considered differences to be significant when *p* < 0.05.

## Figures and Tables

**Table 1:** Oligonucleotides used to assemble genetic constructs for miRNA gene editing via DNA double-strand breaks (LentiCRISPRv2GFP) and subsequent analysis of the edited genomic DNA.

Name	Sequence (5′->3′)	Tm, °C	Cut-Out DNA Fragment Amplicon Length, bp
miR-21-gRNA-site1-f	CACCGATGGAGATGTCACGATGGT	-	498
miR-21-gRNA-site1-r	AAACACCATCGTGACATCTCCATC		
miR-21-gRNA-site2-f	CACCGCTAAGTCAGCCTCTAGTCG		
miR-21-gRNA-site2-r	AAACCGACTAGAGGCTGACTTAGC		
miR-29c-gRNA-site1-f	CACCGAAATCGGTTATGATGTAGG	-	518
miR-29c-gRNA-site1-r	AAACCCTACATCATAACCGATTTC		
miR-29c-gRNA-site2-f	CACCGACTCAAGTGGCAAGAGGAG		
miR-29c-gRNA-site2-r	AAACCTCCTCTTGCCACTTGAGTC		
mir-21-test-f	ACTTGTTCATTTTGTTTTGCTTGG	59.5	885
mir-21-test-r	ACGTATCAATTAGACCTTCAACCTA		
mir-29c-test-f	GAACAGCACTACATTTCAGCAAA	58.0	906
mir-29c-test-r	TGGAAGCTGGTTTCACATGGT		

**Table 2:** Oligonucleotides used to assemble genetic constructs for miRNA gene editing via DNA single-strand breaks (LentiCRISPRD10Av2-GFP) and subsequent analysis of the edited genomic DNA.

Name	Sequence (5′->3′)	Tm, °C	Cut-Out DNA Fragment Amplicon Length, bp
miR-21-gRNAn-site1-f	CACCGTCATGGCAACACCAGTCGA	-	498
miR-21-gRNAn-site1-r	AAACTCGACTGGTGTTGCCATGAC		
miR-21-gRNAn-site2-f	CACCGGATAAGCTACCCGACAAGG		
miR-21-gRNAn-site2-r	AAACCCTTGTCGGGTAGCTTATCC		
miR-29c-gRNAn-site1-f	CACCGGTCTAGCACCATTTGAAAT	-	518
miR-29c-gRNAn-site1-r	AAACATTTCAAATGGTGCTAGACC		
miR-29c-gRNAn-site2-f	CACCGGGTCAGCCTGTGTAAGAGA		
miR-29c-gRNAn-site2-r	AAACTCTCTTACACAGGCTGACCC		
mir-21-nick-test-f	GGAGAGAATTCTAACCCAGTTTTCTTGCCGT	59.5	885
mir-21-nick-test-r	GGAGAGGTACCTCAAAACCCACAATGCAGCTTAG		
mir-29c-nick-test-f	GGAGAGAATTCCAGGACCCACTTCTTATCATCAC	58.0	906
mir-29c-nick-test-r	GGAGAGGTACCTTGACTCCTAGCAGCCATCAC		

## Data Availability

Data are available on request.

## Supplementary Materials

The following supporting information can be downloaded at: https://www.mdpi.com/article/10.3390/ncrna9050049/s1, Figure S1: The results of sequencing of pIX gene within HEK293T genome. Approximately 30% of clones (of more than 100) revealed scarless deletions of approximately 770 bp between the WT CRISPR/Cas9 double-strand cut sites (clones #1, #6, #8 are presented); Figure S2: Summary results of analysis of the clones # 21.C1/C4, 21.6, 21.7. *—TIDE: Tracking of Indels by Decomposition, **—*p* < 0.05 (vs. appropriate control groups, *t*-test), *n* = 3; Figure S3: Summary results of analysis of the clones # 29c.C1/C4, 29c.4, 29c.6, 29c.10 and 29c.11. *—TIDE: Tracking of Indels by Decomposition, **—insufficient cell mass for clones 29c.6 and 29c.10 for reverse transcription was obtained, because they stopped dividing and died. ***—*p* < 0.05 (vs. appropriate control groups, *t*-test), *n* = 3; Figure S4: Summary results of analysis of the clones # 29c.12, 29c.16 and 29c.19. *—TIDE: Tracking of Indels by Decomposition, **—*p* < 0.05 (vs. appropriate control groups, *t*-test), *n* = 3; Figure S5: MSC-EV characteristics. Left panel—Immunoblotting for Alix, HSP70, CD63, CD9, CD81 and H2AX in MSC and hMSC-EV lysates. Right panel—Size and concentration analysis of non-concentrated hMSC-EV after 2 days of conditioning (green) and control aliquots of MSC-CM after 30 min of conditioning (blue) by NTA using ZetaView; Figure S6: The average length of aSMA stress fibers in myofibroblast cells treated with EVs obtained from ASC52telo, native or CRISPR/Cas9-modified; units = total area aSMA (pixel)/number of nuclei *—*p* < 0.05, *n* ≥ 4; Table S1. The results of the study of individual ACS52telo clones after editing the hsa-mir-21 and hsa-mir-29c genes (sequencing and real-time PCR).
