# Supplementary material for: Advances and Obstacles in Using CRISPR/Cas9 Technology for Non-Coding RNA Gene Knockout in Human Mesenchymal Stromal Cells
Source: Noncoding RNA. 2023 Aug 24;9(5):49. doi: 10.3390/ncrna9050049 (PMC10514828; doi:10.3390/ncrna9050049)
Supplement: Supplementary file 1 [file ncrna-09-00049-s001.zip › ncrna-2513813-Supplementary.pdf]

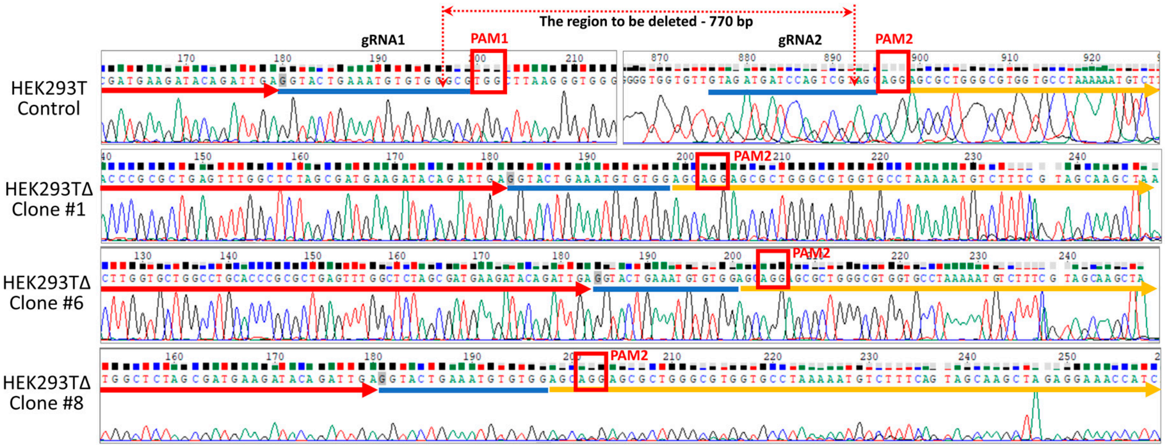

Figure S1. The results of sequencing of pIX gene within HEK293T genome. Approximately 30% of clones (of more than 100) revealed scarless deletions of approximately 770 bp between the WT CRISPR/Cas9 double-strand cut sites (clones #1, #6, #8 are presented).

Clone #	21.C1/C4	21.6				21.7				
Variant	WT	Var1	Var2	Var3	Var4	Var1	Var2	Var3	Var4	Var5
Sequence	5'TGTACCACCTTGTCGGGTAGCTTATCAGACTGATGTTGACTGTTGAATCTCATGGCAACACCAGTCGATGGGCTGTCTGACATTTTGGTATC	5'TTTTTATCAAATCAGACTGATGTTGACTGTTGAATCTCATGGGTTATCAGACTGATGTTGACTGTTGAATCTCATGTCGGGTAGCTTATCAGACTGATGTTGACTGTTGAATCTCATGGGTTATCAGACTGATGTTGACTGTTGAATCTCATGGGTTATCAGACTGATGTTGACTGTTGAATCTCATGTCGGGTAGCTTATCAGACTGATGTTGACTGTTGAATCTCATGGGTTATCAGACTGATGTTGACTGTTGAATCTCATGGCAACACCAGTCGATGGGCTGTCTGACATTTTGGTATC	5'TTTTTATCAAATCAGACTGATGTTGACTGTTGAATCTCATGGGTTATCAGACTGATGTTGACTGTTGAATCTCATGGCGGATAACATAGCTGACTGATGTTGACTGTCGTGTCTCATGGGTTATCAGACTGATGTTGACTGTTGAATCTCATGGGTTATCAGACTGATGTTGACTGTTGAATCTCATGTCGGGTAGCTTATCAGACTGATGTTGACTGTTGAATCTCATGGGTTATCAGACTGATGTTGACTGTTGAATCTCATGGCAACACCAGTCGATGGGCTGTCTGACATTTTGGTATC	5'TGAATCTCATGTCGGGTAGCTTATCAGACTGATGTTGACTGTTGAATCTCATGGGTTATCAGACTGATGTTGACTGTTGAATCTCATGGGTTATCAGACTGATGTTGACTGTTGAATCTCATGTCGGGTAGCTTATCAGACTGATGTTGACTGTTGAATCTCATGGGTTATCAGACTGATGTTGACTGTTGAATCTCATGGCAACACCAGTCGATGGGCTGTCTGACATTTTGGTATC	5'TGTACCACCTTTGACTGTTGAATCTCATGGGTTATCAGACTGATGTTGACTGTTGAATCTCATGGCAACACCAGTCGATGGGCTGTCTGACATTTTGGTATC	5'TGTACCACCATGGAGCTTATCAGACTGATGTTGACTGTTGAATCTCATGGCAACACCACCAGTTGTCCAGACTGATGTTGACTGTTGAATCTCATGGCAACACCAGTTGTCGGGTAGCTTATCAGACTGATGTTGACTGTTGAATCTCATGGCAACACCACCAGTTGTCGGGTAGCTTATCAGACTGATGTTGACTGTTGAATCTCATGGCAACACCAGTCGATGGGCTGTCTGACATTTTGGTATC	5'CTGTACCACCTTGTCGGGTAGCTTATCAGACTGATGTTGACTGTTGAATCTCATGGCAACATCGGGTATGTTGACTGTTGAATCTCATGGCAACATCGGGTAGCTTATCAGACTGATGTTGACTTTATCAGACTGATGTTGACTGTTGAATCTCATGGCAACATCGGGTAGCTTATCACGATGGGCTGTCTGACATTTTGGTATC	5'TGTACCACCTTGTCGGGTAGCTTATCAGACTGATGTTGACTGTTGAATCTCATGGCAACATCGGGTAGCTTATCAGACTGATGTTGACTTTATCAGACTGATGTTGACTGTTGAATCTCATGGCAACATCGGGTAGCTTATCACGATGGGCTGTCTGACATTTTGGTATC	5'ACCACCATGGAGCTTATCAGACTGATGTTGACTGTTGAATCTCATGGCAACACCACCAGTTGTCCAGACTGATGTTGACTGTTGAATCTCATGGCAACACCAGTTGTCGGGTAGCTTATCAGACTGATGTTGACTGTTGAATCTCATGGCAACACCACCAGTTGTCGGGTAGCTTATCAGACTGATGTTGACTGTTGAATCTCATGGCAACACCAGTCGATGGGCTGTCTGACATTTTGGTATC	5'ACCACCATGGAGCTTATCAGACTGATGTTGACTGTTGAATCTCATGGCAACTGTCGGGTAGCTTATCAGACTGATGTTGACTGTTGAATCTCATGGCAACACCACCAGTTGTCGGGTAGCTTATCAGACTGATGTTGACTGTTGAATCTCATGGCAACACCAGTCGATGGGCTGTCTGACATTTTGGTATC
Predicted pri-miRNA secondary structures	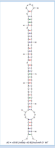	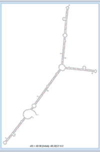		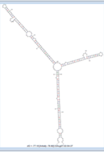	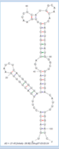	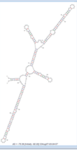	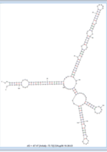	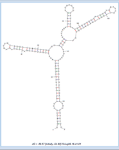	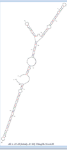	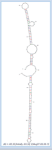
Predicted miRNA-like sequences	**5'UAGCUUAUCAGACUGAUGUUGA**<br>**5'CAACACCAGUCGAUGGGCUGU**<br>5'CAGUCGAUGGGCUGUCUGACAU<br>5'UGUCGGGUAGCUUAUCAGACUG	5'GGGUUAUCAGACUGAUGUUGAC<br>5'GUUGAAAUCUCAUGUCGGGUAG<br>5'GUCGGGUAGCUUAUCAGACUGA<br>5'CAGUCGAUGGGCUGUCUGACAU	5'UCAAAUCAGACUGAUGUUGACU<br>5'GGUUAUCAGACUGAUGUUGAC<br>5'UGGCAACACCAGUCGAUGGGCU<br>5'UUAUCAGACUGAUGUUGACUGU	5'GACUGAUGUUGACUGUUGAAUC<br>5'UGACUGUUGAAUCUCAUGGGUU<br>5'AUCUCAUGGUUAUCAGACUGAU<br>5'UCAUGUCGGGUAGCUUAUCAGA<br>5'GGCAACACCAGUCGAUGGGCUG<br>5'UAUCAGACUGAUGUUGACUGU	—	5'UAUCAGACUGAUGUUGACUGUU<br>5'GCAACACCAGUCGAUGGGCUG	—	—	5'CAACACCAGUUGUCGGGUAGCU<br>5'AGUUGUCCAGACUGAUGUUGAC<br>5'CAACACCAGUCGAUGGGCUGUC<br>5'AGCUUAUCAGACUGAUGUUGAC	5'AGGCAACACCAGUCGAUGGGGU<br>5'UAUCAGACUGAUGUUGACUGU
Variant Frequency	100%	30%	20%	40%	10%	10%	10%	30%	20%	30%
TIDE* analysis	-	del 75, ins 147				no intact alleles				
Real-Time PCR for hsa-miR-21-5p	n.d.	↓**				↓**				

Figure S2. Summary results of analysis of the clones # 21.C1/C4, 21.6, 21.7. *—TIDE: Tracking of Indels by Decomposition, **—p < 0.05 (vs. appropriate control groups, t-test), n = 3.

Clone #	29c.C1/C4	29c.4				29c.6**		29c.10**		29c.11	
Variant	WT	Var1	Var2	Var3	Var4	Var1	Var2	Var1	Var2	Var1	Var2
Sequence	5'CCACTGGCCCATCTCTTACACAGGCTGACCGATTTCTCCTGGTGTTCAGAGTCTGTTTTTGTCTAGCACCATTTGAAATCGGTTATGATGTAGGGGGAAAAGCAGCAG	5'CCACTGGCCCATCTCTTACACAGGCTGACCGATTTCTCCTGGTGTTCAGAGTCTGTTTTTGTCTAGCACCAGGCTGACCGATTTCTCCTGGTGTTCAGAGTCTGTTTTTGTCTAGCACCATTTGAAATCGGTTATGATGTAGGGGGAAAAGCAGCAG	5'CTGGCCCATCTCTTACACAGGCTGACCGATTTCTCCTGGTGTTCAGAGTCTGTTTTTGTCTAGCACCCTGACCGATTTCTCCTGGTGTTCAGAGTCTGTTTTTGTCTAGCACCATTTGAAATCGGTTATGATGTAGGGGGAAAAGCAGCAG	5'CTGGCCCATCTCTTACACAGGCTGACCGATTTCTCCTGGTGTTCAGAGTCTGTTTTTGTCTAGCACCTGTCTAGCACCCTGACCGATTTCTCCTGGTGTTCAGAGTCTGTTTTTGTCTAGCACCATTTGAAATCGGTTATGATGTAGGGGGAAAAGCAGCAG	5'CTGGCCCATCTCTTACACAGGCTGACCGATTTCTCAGAGTCTGTTTTTGTCTAGCACCCTGACCGATTTCTCCTGGTGTTCAGAGTCTGTTTTTGTCTAGCACCATTTGAAATCGGTTATGATGTAGGGGGAAAAGCAGCAG	5'CCACTGGCCCATCTCTTACACAGGCTGACCGATTTCTCCTGGTGTTCAGAGTCTGTTTATGATGTAGGGGGAAAAGCAGCAG	5'CCACTGGCCCATCTCTTACACAGGCTGACCGATTTCTCCTGGTGTTCAGAGTCTGTTTCTCCTGGTGTTCAGAGTCTGTTTTTGTCTAGCACATGATGTAGGGGGAAAAGCAGCAG	5'CCACTGGCCCATCTCTTACACAGGCTGACCGATTTCTCCTGGTGTTCAGAGTCTGTATGATGTAGGGGGAAAAGCAGCAG	5'CCACTGGCCCCGATTTCTCCTGGTGTTCAGAGTCTGTTTTTGTCTAGCACATCTCTTACACAGGCTGACCGATTTCTCCTGGTGTTCAGCACATGATGTAGGGGGAAAAGCAGCAG	5'CCACTGGCCCATCTCAGGCTGACCGATTTCTCCTGGTGTTCAGAGTCTGTTTTTGTCTACACAGGCTGACCGATTTCTCCTGGTGTTCAGAGTCTGTTTTTGTCTAGCACCATTTGAAATCGGTTATGATGTAGGGGGAAAAGCAGCAG	5'CCACTGGCCCATCTCAGGCTGACCGATTTCTCCTGGTGTTCAGAGTCTGTTTTTGTCTTACACAGGCTGACCGATTTCTCCTGGTGTTCAGAGTCTGTTTTTGTCTAGCACCATTTGAAATCGGTTATGATGTAGGGGGAAAAGCAGCAG
Predicted pri-miRNA secondary structures	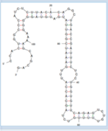	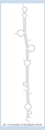	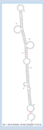	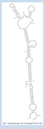	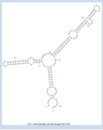	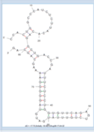	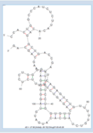	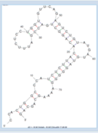	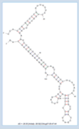	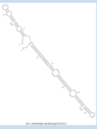	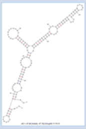
Predicted miRNA-like sequences	**5'UGACCGAUUUCUCCUGGUGUUC**<br>**5'UAGCACCAUUUGAAAUCGGUUA**	5'CUUACACAGGCUGACCGAUUUC<br>5'AAUCGGUUAUUGAUGUAGGGGG	5'CUUACACAGGCUGACCGAUUUC<br>5'AAUCGGUUAUUGAUGUAGGGGG	5'CUUACACAGGCUGACCGAUUUC<br>5'AAUCGGUUAUUGAUGUAGGGGG	5'UGACCGAUUUUCUCCUGGUGUU<br>5'UAGCACCAUUUGAAAUCGGUUA	—	—	—	5'UGAUGUAGGGGGAAAAGCAGC<br>5'GUCUGUUUUUGUCUAGCACAUC	—	—
Variant Frequency	100%	20%	20%	40%	20%	50%	50%	50%	50%	50%	50%
TIDE* analysis	-	del 27, ins 40				del 19, del 26		ins 8, del 28		ins 41, ins 46	
Real-Time PCR for hsa-miR-29c-3p	-	n.d.				n.t.**		n.t.**		↓***	

Figure S3. Summary results of analysis of the clones # 29c.C1/C4, 29c.4, 29c.6, 29c.10 and 29c.11. *—TIDE: Tracking of Indels by Decomposition, **—insufficient cell mass for clones 29c.6 and 29c.10 for reverse transcription was obtained, because they stopped dividing and died. ***—p < 0.05 (vs. appropriate control groups, t-test), n = 3.

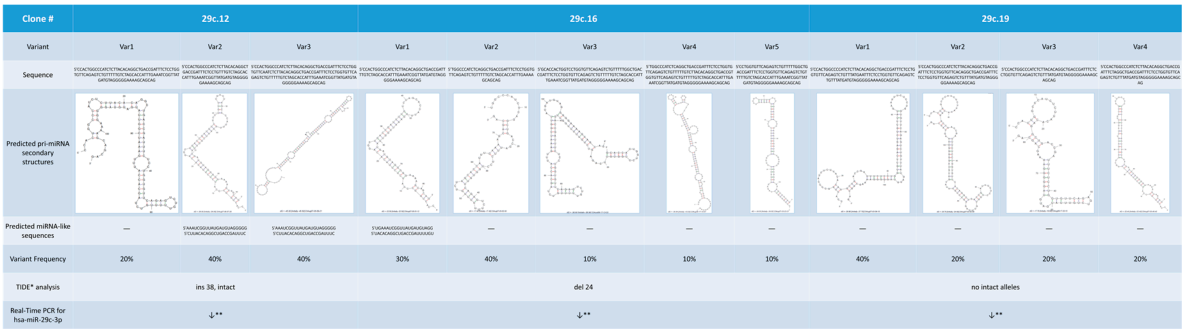

Clone #	29c.12			29c.16					29c.19			
Variant	Var1	Var2	Var3	Var1	Var2	Var3	Var4	Var5	Var1	Var2	Var3	Var4
Sequence	5'CCACTGGCCCATCTCTTACACAGGCTGACCGATTTCTCCTGGTGTTCAGAGTCTGTTTTTGTCTAGCACCATTTGAAATCGGTTATGATGTAGGGGGAAAAGCAGCAG	5'CCACTGGCCCATCTCTTACACAGGCTGACCGATTTCTCCTGTTTGTCTAGCACCATTTGAAATCGGTTATGATGTAGGGGGAAAAGCAGCAG	5'CCACTGGCCCATCTCTTACACAGGCTGACCGATTTCTCCTGGTGTTCAATCTCTTACACAGGCTGACCGATTTCTCCTGGTGTTCAGAGTCTGTTTTTGTCTAGCACCATTTGAAATCGGTTATGATGTAGGGGGAAAAGCAGCAG	5'CCACTGGCCCATCTCTTACACAGGCTGACCGATTTTGTCTAGCACCATTTGAAATCGGTTATGATGTAGGGGGAAAAGCAGCAG	5'TGGCCCATCTCAGGCTGACCGATTTCTCCTGGTGTTCAGAGTCTGTTTTTGTCTAGCACCATTTGAAAAGCAGCAG	5'GCACCACTGGTCCTGGTGTTCAGAGTCTGTTTTTGGCTGACCGATTTCTCCTGGTGTTCAGAGTCTGTTTTTGTCTAGCACCATTTGAAATCGGTTATGATGTAGGGGGAAAAGCAGCAG	5'TGGCCCATCTCAGGCTGACCGATTTCTCCTGGTGTTCAGAGTCTGTTTTTGTCTTACACAGGCTGACCGTGGTGTTCAGAGTCTGTTTTTGTCTAGCACCATTTGAAATCGGTTATGATGTAGGGGGAAAAGCAGCAG	5'CCTGGTGTTCAGAGTCTGTTTTTGGCTGACCGATTTCTCCTGGTGTTCAGAGTCTGTTTTTGTCTAGCACCATTTGAAATCGGTTATGATGTAGGGGGAAAAGCAGCAG	5'CCACTGGCCCCATCTCTTACACAGGCTGACCGATTTCTCCTGGTGTTCAGAGTCTGTTTATGAATTTCTCCTGGTGTTCAGAGTCTGTTTATGATGTAGGGGGAAAAGCAGCAG	5'CCACTGGCCCATCTCTTACACAGGCTGACCGATTTCTCCTGGTGTTCACAGGCTGACCGATTTCTCCTGGTGTTCAGAGTCTGTTTATGATGTAGGGGGAAAAGCAGCAG	5'CCACTGGCCCATCTCTTACACAGGCTGACCGATTTCTCCTGGTGTTCAGAGTCTGTTTATGATGTAGGGGGAAAAGCAGCAG	5'CCACTGGCCCATCTCTTACACAGGCTGACCGATTTCTAGGCTGACCGATTTCTCCTGGTGTTCAGAGTCTGTTTATGATGTAGGGGGAAAAGCAGCAG
Predicted pri-miRNA secondary structures												
Predicted miRNA-like sequences	—	5'AAAUCGGUUAUGAUGUAGGGGG 5'CUUACACAGGCUGACCGAUUUC	5'AAAUCGGUUAUGAUGUAGGGGG 5'CUUACACAGGCUGACCGAUUUC	5'UGAAAUCGGUUAUGAUGUAGG 5'UACACAGGCUGACCGAUUUUGU	—	—	—	—	—	—	—	—
Variant Frequency	20%	40%	40%	30%	40%	10%	10%	10%	40%	20%	20%	20%
TIDE* analysis	ins 38, intact			del 24					no intact alleles			
Real-Time PCR for hsa-miR-29c-3p	↓**			↓**					↓**			

Figure S4. Summary results of analysis of the clones # 29c.12, 29c.16 and 29c.19. *—TIDE: Tracking of Indels by Decomposition, **—$p < 0.05$ (vs. appropriate control groups, *t*-test), $n = 3$.

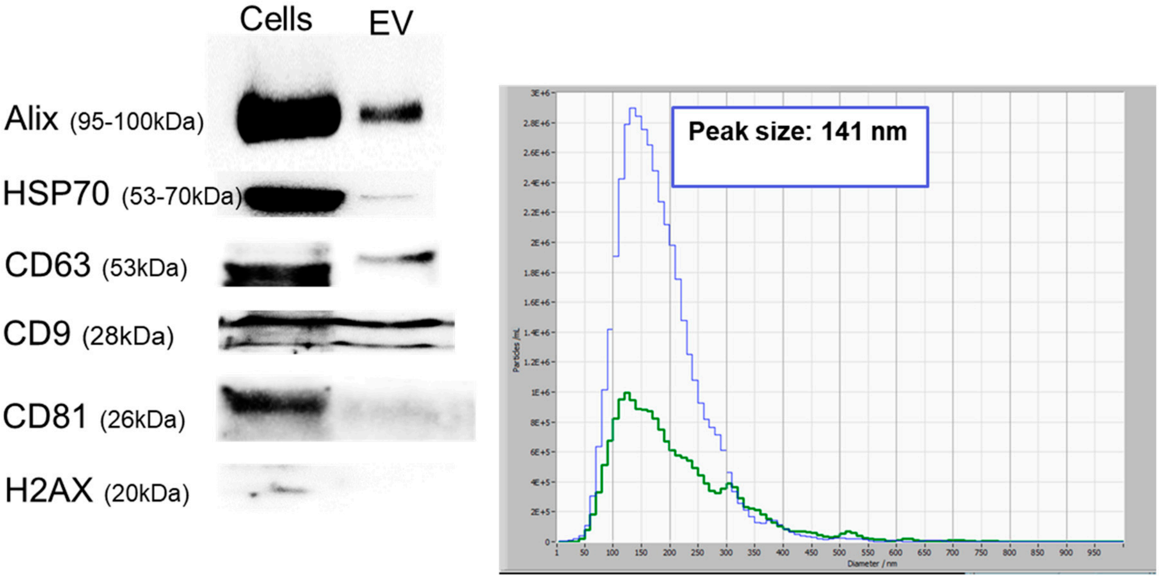

Figure S5. MSC-EV characteristics. Left panel—Immunoblotting for Alix, HSP70, CD63, CD9, CD81 and H2AX in MSC and hMSC-EV lysates. Right panel—Size and concentration analysis of non-concentrated hMSC-EV after 2 days of conditioning (green) and control aliquots of MSC-CM after 30 min of conditioning (blue) by NTA using ZetaView.

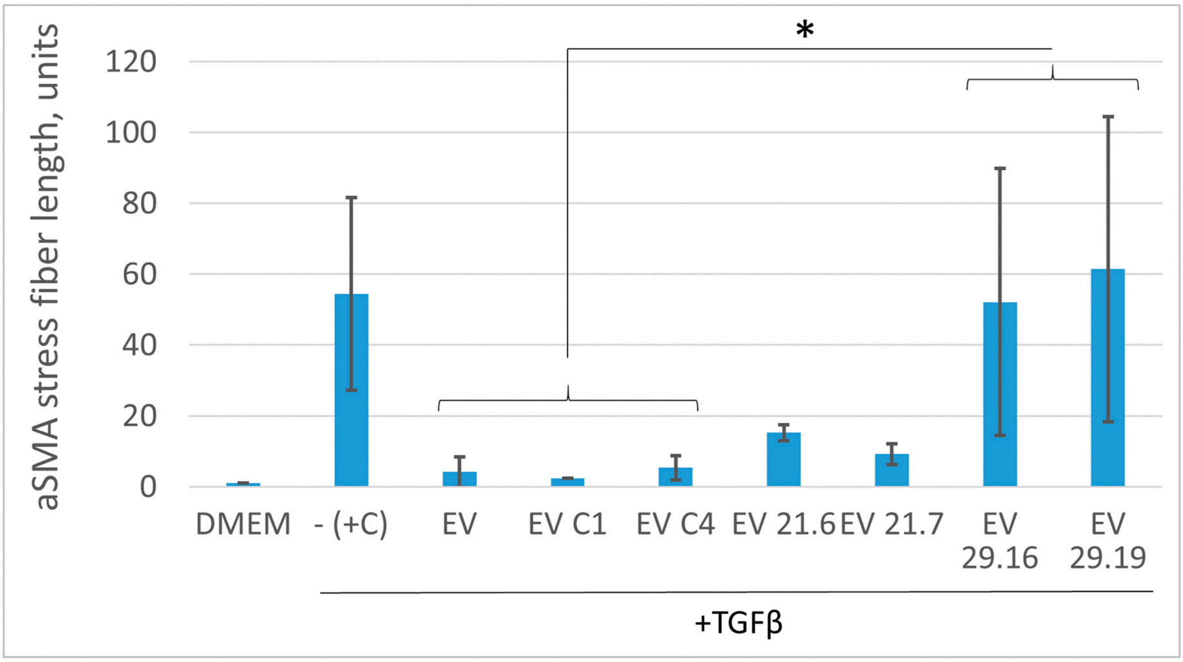

Figure S6. The average length of aSMA stress fibers in myofibroblast cells treated with EVs obtained from ASC52telo, native or CRISPR/Cas9-modified; units = total area aSMA (pixel)/number of nuclei *—$p < 0.05$, $n \geq 4$.

Table S1. The results of the study of individual ACS52telo clones after editing the hsa-miR-21 and hsa-miR-29c genes (sequencing and real-time PCR)

hsa-miR21			
**Clone #**	**TIDE***	**Plasmid library sequencing**	**Real-Time PCR**
1	intact	n.t.	n.d.
2	intact, del 26	n.t.	n.t.
3	intact	n.t.	n.d.
4	intact	n.t.	↑
5	intact	n.t.	n.t.
6	del 75, ins 147	edited	↓**
7	no intact alleles	edited	↓**
8	intact	n.t.	n.d.

hsa-miR29C			
**Clone #**	**TIDE***	**Plasmid library sequencing**	**Real-Time PCR**
1	ins 37-42, 44-47	edited	n.d.
2	ins 47, intact	n.t.	n.d.
3	del 27, ins 26	n.t.	↑
4	del 27, ins 40	edited	n.d.
5	del 300, ins 9	edited	n.t.
6	del 19, del 26	edited	n.t.***
7	ins 9, ins 16	edited	↓**
8	ins 9, ins 29	edited	n.d.
9	ins 9, ins 29	n.t.	n.t.
10	ins 8, del 28	edited	n.t.***
11	ins 41, ins 46	edited	↓**

12	ins 38, intact	edited	↓**
13	ins 9, ins 37	n.t.	n.d.
14	no intact alleles	edited	n.d.
15	ins 9, ins 37	n.t.	n.d.
16	del 24	edited	↓**
17	no intact alleles	edited	↓**
18	ins 9, ins 34	edited	↓**
19	no intact alleles	edited	↓**

* - TIDE: Tracking of Indels by Decomposition (Brinkman EK, Chen T, Amendola M, van Steensel B. Easy quantitative assessment of genome editing by sequence trace decomposition. Nucleic Acids Res. 2014 Dec 16;42(22):e168. doi: 10.1093/nar/gku936), http://shinyapps.datacurators.nl/tide/ (last access - August 2nd, 2023).

** - $p < 0.05$ (vs appropriate Control groups, t-test), n = 3.

*** - not enough cell mass of clones 29c.6 and 29c.10 for reverse transcription was obtained, because they stopped dividing and died.

n.t. - not tested

n.d. - no difference to the appropriate Control group (Real-Time PCR)

For the clones marked green (decrease expression of an appropriate miRNA, or a prominent genome-editing outcome according to TIDE) plasmid libraries were prepared and sequenced.
